# lncRNA DQ786243 promotes hepatocellular carcinoma cell invasion and proliferation by regulating the miR-15b-5p/Wnt3A axis

**DOI:** 10.3892/mmr.2022.12912

**Published:** 2022-12-09

**Authors:** Zewei Lin, Jikui Liu

Mol Med Rep 23: 318, 2021; DOI: 10.3892/mmr.2021.11957

Following the publication of this article, an interested reader drew to the authors’ attention that the primer sequences written for lncRNA DQ786243 and miR-15b-5p on p. 2 in the study were incorrect. Upon requesting an explanation of these errors from the authors, they realized that, regarding the sequence of the reverse primer for lncRNA DQ786243, three nucleotides were omitted from its 3’-end [the sequence of this primer on p. 2, right-hand column, line 25 should have been written as 5’-CTTCTGCTGGGCTGTTGAGTG-3’ (with the omitted nucleotides highlighted in bold)]. Regarding the primers of miR-15b-5p, the authors used the mature miR-15b-5p sequence as the forward primer; however, they inadvertently overlooked replacing U with T in the description of the forward primer of miR-15b-5p, and therefore the sequence of the forward primer of miR-15b-5p on line 27 should have been written as 5’-**T**AGCAGCACA**T**CA**T**GG**TTT**ACA-3’. Moreover, a universal reverse primer was used for the reverse primer of miR-15b-5p, as provided by the kit [specifically, the authors used an Mir-X miRNA qRT-PCR TB Green Kit (Takara Bio USA, Inc.) for detecting the expression of miR-15b-5p, and the reverse primer was supplied in the kit]; however, a different primer sequence used in the authors’ lab was erroneously written as the reverse primer of miR-15b-5p in the manuscript. Finally, note that the title was published with a typographical error: “miR-15p-5p” in the title should have been written as “miR-15**b**−5p”, as appeared elsewhere throughout the paper, and the corrected title is presented above.

The authors are grateful to the Editor of *Molecular Medicine Reports* for allowing them the opportunity to publish this corrigendum, and all the authors agree with its publication. The authors also regret the inconvenience that these mistakes have caused.

